# High-Resolution Analysis of the 5′-End Transcriptome Using a Next Generation DNA Sequencer

**DOI:** 10.1371/journal.pone.0004108

**Published:** 2009-01-01

**Authors:** Shin-ichi Hashimoto, Wei Qu, Budrul Ahsan, Katsumi Ogoshi, Atsushi Sasaki, Yoichiro Nakatani, Yongjun Lee, Masako Ogawa, Akio Ametani, Yutaka Suzuki, Sumio Sugano, Clarence C. Lee, Robert C. Nutter, Shinichi Morishita, Kouji Matsushima

**Affiliations:** 1 Department of Molecular Preventive Medicine, Graduate School of Medicine, The University of Tokyo, Tokyo, Japan; 2 Department of Computational Biology, Graduate School of Frontier Sciences, The University of Tokyo, Kashiwa, Japan; 3 Department of Medical Genome Science, Graduate School of Frontier Science, The University of Tokyo, Kashiwa, Japan; 4 Applied Biosystems, Foster City, California, United States of America; Purdue University, United States of America

## Abstract

Massively parallel, tag-based sequencing systems, such as the SOLiD system, hold the promise of revolutionizing the study of whole genome gene expression due to the number of data points that can be generated in a simple and cost-effective manner. We describe the development of a 5′–end transcriptome workflow for the SOLiD system and demonstrate the advantages in sensitivity and dynamic range offered by this tag-based application over traditional approaches for the study of whole genome gene expression. 5′-end transcriptome analysis was used to study whole genome gene expression within a colon cancer cell line, HT-29, treated with the DNA methyltransferase inhibitor, 5-aza-2′-deoxycytidine (5Aza). More than 20 million 25-base 5′-end tags were obtained from untreated and 5Aza-treated cells and matched to sequences within the human genome. Seventy three percent of the mapped unique tags were associated with RefSeq cDNA sequences, corresponding to approximately 14,000 different protein-coding genes in this single cell type. The level of expression of these genes ranged from 0.02 to 4,704 transcripts per cell. The sensitivity of a single sequence run of the SOLiD platform was 100–1,000 fold greater than that observed from 5′end SAGE data generated from the analysis of 70,000 tags obtained by Sanger sequencing. The high-resolution 5′end gene expression profiling presented in this study will not only provide novel insight into the transcriptional machinery but should also serve as a basis for a better understanding of cell biology.

## Introduction

Genome-wide analysis of gene expression in different cell subpopulations provides insights into many aspects of developmental biology and physiology. Although established functional genomic technologies, such as DNA arrays and serial analysis of gene expression (SAGE), can identify coding and noncoding RNA transcripts, identification of genes across the whole genome is still problematic. Most unique transcripts are expressed at low levels[1], [2] and fundamental cellular mechanisms cannot be identified by the limited number of genes analyzed per study. In addition, the heterogeneity of transcriptional start sites in each gene region is not still well characterized. Currently, expression profiling is usually carried out by hybridization to microarrays. This approach, while immensely useful, is not very quantitative, as it typically yields relative rather than absolute mRNA abundance, and the results are difficult to compare across different microarray platforms.

On the other hand, SAGE[1], [3] and 5′-end SAGE technology (5′SAGE)[4] provide digital readouts of the number of mRNA molecules in a sample by sequencing short sequence tags. While the use of the SAGE techniques has been limited by complicated protocols for sample preparation and the scale of the subsequent sequencing, these limitations might be overcome by combining these techniques with massively parallel sequencing technologies. 5′ SAGE enables genome-wide identification of transcription start sites (TSSs) in addition to quantification of mRNA transcripts. Genome-wide analysis of small DNA sequence tags from RNA molecules using next generation DNA sequencing systems, such as Roche/454 [5], Illumina/Solexa [6], [7], PMAGE system [8], and SOLiD system[9], [10] provides a 2–3 order of magnitude increase in the amount of sequence that can be cost-effectively generated relative to traditional technology. In this study, by combining the newest sequencing method, “Sequencing by oligonucleotide ligation and detection” (SOLiD™), with 5′SAGE, we have developed a 5′-end SOLiD technology (5′SOLiD) that can be used to identify transcriptional start sites and quantitative transcript levels in any cell type in a comprehensive fashion.

Elucidation of the role of epigenetic regulation of the genome will be achieved by a detailed characterization of the gene expression machinery. Epigenetic processes are essential for normal development and cell differentiation in all species[11]. Chromatin modifications are known to impose epigenetic controls on gene expression without any requirement for changes in DNA sequence. Recently, it has been hypothesized that an imbalance of epigenetic alterations such as histone deacetylation and DNA methylation in the promoter regions of cancer-related genes plays a crucial role in the development of cancer [12]–[14]. An important factor in tumor development may be the epigenetic effects on tumor suppressor genes. However, how epigenetic drugs mediate their effects in the whole genome is poorly understood. In this study, 5′ SOLiD technology was used to study whole genome gene expression of a colon cancer cell line, HT-29, treated with 5Aza as a test sample.

## Results

### Development of 5′SOLiD sequencing technology

**Figure 1:**
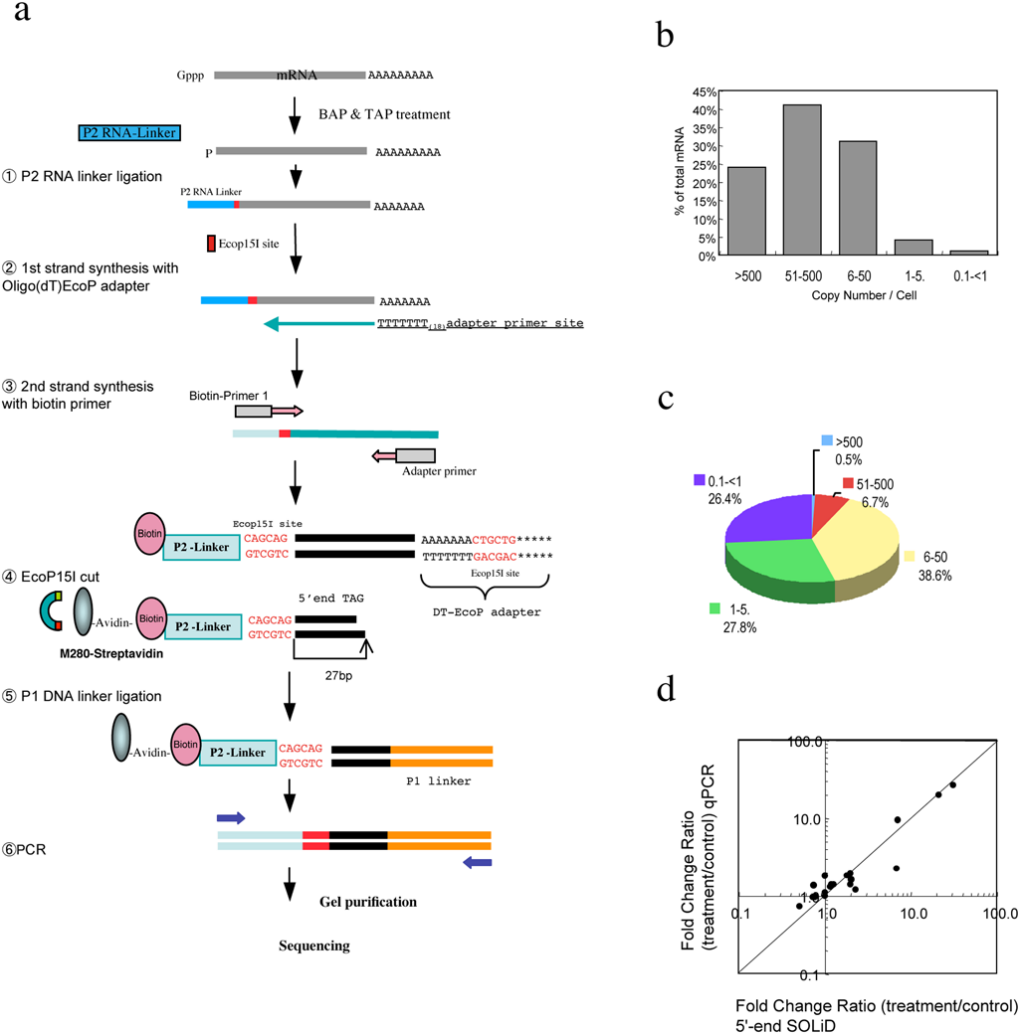
Schematic depicting the 5′-end library construction for SOLiD sequencing (a) and the transcript copy number and abundance in libraries prepared from HT-29 cells, either untreated or treated with 5Aza. Total expressed mRNA (b). The frequency denotes the category of expression level as determined by the number of transcript copies per cell. Unique genes represent the total number of unique genes that corresponded to the RefSeq dataset (c). 5 copies = 100 tags/6 million tags, since human cells are predicted to contain 300,000 mRNA molecules. (d) Validation of the 5′SOLiD analysis using qPCR. Comparison of the 5′SOLiD tag profiling gene expression data with qPCR. The mRNAs corresponding to 40 genes in cells treated with 5Aza were quantified using qPCR. The ratio of mRNA abundances in 5Aza-treated versus control cells determined by this method was compared to the corresponding ratios determined using 5′SOLiD data. The logarithmic values of these ratios were plotted. The Y = X line with a slope of 1 is the expected line when both platforms have identical expression patterns.

The tag length used in 5′SAGE technology (19 bp) renders it difficult to identify precisely the genome position of some of the sequence tags. We therefore developed a method to improve 5′SAGE by the generation of 27 mer sequence tags combined with massively parallel sequencing. Figure 1a shows the process used for 5′-end library construction for SOLiD sequencing. cDNA was synthesized after ligating an RNA linker including an EcoP15I site, which cleaves 27 bp downstream of the recognition site leaving a 5′ overhang of two bases,[15] to the 5′ end of mRNAs using the oligo-cap method[16]. The full-length, double-strand cDNA with a biotinylated 5′end was then digested with EcoP15I. A 27 bp fragment from the 5′end of the cDNA was then purified using avidin-bound magnetic beads. DNA fragments were ligated with sequencing adaptors and amplified by PCR, followed by sequencing with the SOLiD system.

### Summary of sequence tags

We conducted 5′-end sequencing of mRNAs isolated from human colon adenocarcinoma HT-29 cells treated the epigenetic agent, 5Aza, a potent inhibitor of DNA methylation. Using the SOLiD platform, we generated approximately 62 million 25 bp reads (Table 1). Erroneous sequence tags were eliminated using quality values before alignment. The quality value (QV) is a well-known indicator for evaluation on error probability of a base. It is assigned by the SOLiD base (color) caller and estimates the probability each base is called correctly. The probability of a base being wrong *p* is given by *p* = 1/10^QV/10^ [17]. We removed any read which had 5 or more of the first 22 bases with a QV <9. Although massively parallel sequencing technologies achieve dramatically higher throughputs than capillary sequencing, the ability of the short read lengths to map uniquely to a reference sequence is more sensitive to base calling errors. There is the flexibility to choose a stricter or looser QV criterion depending on how much the user wants to reduce the risk mentioned above. Filtering low quality reads yielded ∼35.9 million reads, and ∼23.0 million (64%) of these were aligned to the genome with at most two mismatches in order to cope with sequencing errors and SNPs. Among mapped reads, ∼16.4 million (72%) were uniquely anchored on the genome. The ratios of unique reads aligned with zero, one, and two mismatches were 36%, 32%, and 32%, respectively. 73% of unique reads were located within 500 bases of the public representative transcription start sites (TSSs) of well-annotated protein-coding genes in the RefSeq database. Among the remaining 27% of unique reads, 11% were localized to RefSeq gene-coding regions within the genome, while 16% may represent small RNAs and annotated genes. In this study, we further analyzed the RefSeq associated reads. The 30 most highly expressed transcripts identified here in HT-29 cells are shown in Table S1. The most highly expressed genes in the untreated (control) cells encoded ribosomal proteins and ornithine decarboxylase antizyme 1. The frequency of these transcripts in the library prepared from 5 Aza treated-cells was similar to the level in the library prepared from the control cells.

**Table 1:** Sequencing Summary.

	Sequenced Tags	Used Tags (A)	Mapped Tags (B)	% (B/A)	Unique Tags (C)	% (C/B)	Unique tags in RefSeq TSSs (D)	% (D/C)
Control	29,231,644	16,050,987	9,980,657	62%	7,062,060	71%	5,110,167	72%
5Aza	32,629,872	19,865,139	13,037,914	66%	9,373,606	72%	6,981,749	74%
Total	61,861,516	35,916,126	23,018,571	64%	16,435,666	72%	12,091,916	73%

	Unique Tags (C)	No mismatches (E)	% (E/C)	One mismatch (F)	% (E/C)	Two mismatches (G)	% (E/C)
Control	7,062,060	2,268,809	32%	2,325,304	33%	2,467,947	35%
5Aza	9,373,606	3,771,377	40%	2,879,864	31%	2,722,365	29%
Total	16,435,666	6,040,186	36%	5,205,168	32%	5,190,312	32%

Unique tags were aligned to a position unambiguously. Unique tags in TSSs present numbers of unique tags mapped to the regions within 500 bases from the representative TSSs of genes in the RefSeq database. Unique tags are categorized into three groups according to the number of mismatches in individual alignments.

In order to assess the reproducibility of the system, we compared the unique reads mapping to RefSeq genes isolated from two independent sequence runs from the same library. These data showed that data generated from technical replicates of the 5′SOLiD library were highly reproducible (Pearson's correlation coefficient *r*>0.99) (Figure S1a, b). A high degree of reproducibility of the sequence data generated from independent analyses of the same library is essential for accurate detection of subtle changes in gene expression.

### Copy number and abundance of protein coding mRNA-associated transcripts

We next calculated the number of genes expressed in a single cell type. As human cells contain approximately 300,000 mRNA molecules[1], we evaluated RefSeq associated genes among the 5′SOLiD tags. The level of expression of these genes ranged from ∼0.02 to ∼4,704 transcripts per cell. The unique transcript tags identified in the control and 5Aza library at more than 0.1 copies/cell corresponded to approximately 14,900 and 14,500 RefSeq associated genes, respectively (Table 2). Only transcripts present at greater than 3∼5 copies/cell can be detected by regular SAGE sequencing or DNA array methods [18]. These RNAs represent 95% of the total expressed mRNAs (Figure 1b). However, genes expressed at less than 5 copies /cell accounted for about 60% of the total expressed genes in this study (Figure 1c). Similar numbers of expressed genes and a similar range of copy number were observed in control and 5Aza treated cells.

**Table 2:** Copy number and transcripts abundance in each library.

Copy/cell	Cont (Unique genes)		Mass fraction mRNA	5Aza (Unique genes)		Mass fraction mRNA
>500	68	0%	24%	65	0%	24%
51–500	1016	7%	41%	1046	7%	41%
6–50	5666	38%	30%	5609	39%	30%
1–5.	4012	27%	4%	3913	27%	4%
0.1–<1	4151	28%	1%	3904	27%	1%
Total	14913	100%		14537	100%	

Frequency denotes the category of expression level analyzed in transcript copies per cell in each librasiry. Unique genes represent a total number of unique genes hit to the RefSeq sequencing. An estimate of about 300,000 transcripts per cell was used to concert the abundances to copies per cell.

### Comparison of gene expression patterns between 5′SAGE and 5′SOLiD

**Figure 2:**
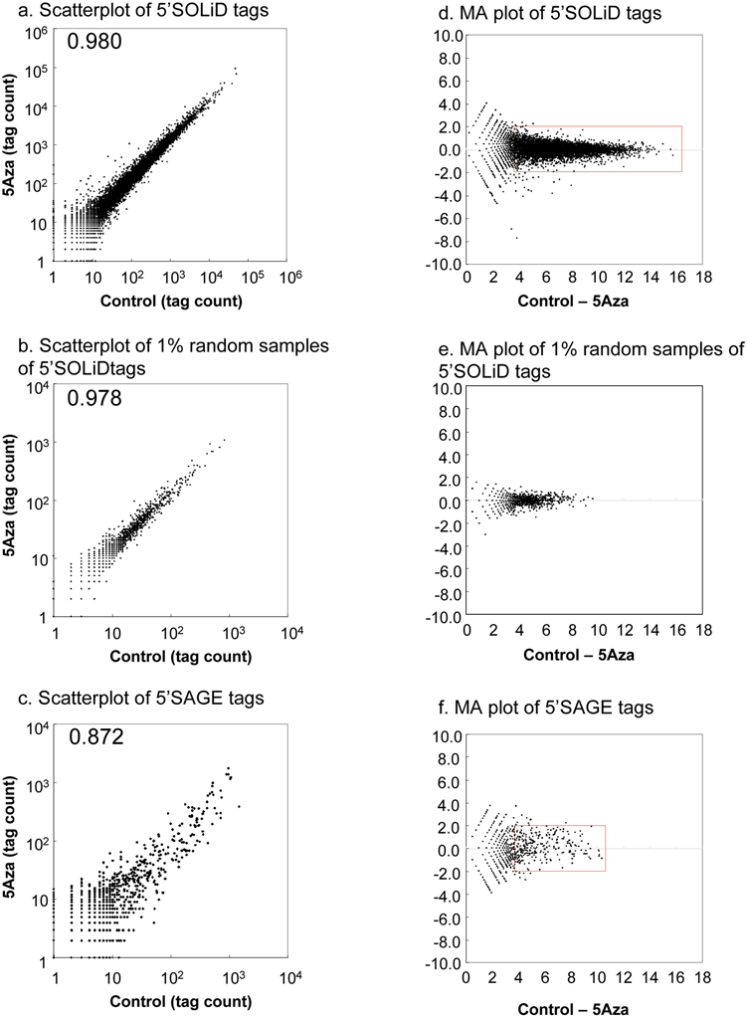
Comparison of gene expression patterns between 5′SOLiD and 5′SAGE. These data show a scatter plot of unique transcripts identified by SOLiD sequencing (a) and random samples of 5′SOLiD tags (b) 5′SAGE tag sequencing(c) of libraries prepared from RNA isolated from HT29 cells treated with 5Aza. Individual 5′-end tags are associated with the human gene in which the tag originated. In the graph, each dot represents one gene, and its x and y coordinates indicate the numbers of 5′-end tags associated with the gene in each library (a–c). The number represents a comparison of the Pearson Correlation coefficient between two libraries. (d)–(f), MA plots are calculated from the pair of the number of 5′SOLiD tags and the number of 5′SAGE tags that are associated with a gene. X and Y values are translated into A and M values according to the following formulas: A = ½ (log_2_X+log_2_Y), M = log_2_X−log_2_Y.

To evaluate the dynamic range and sensitivity of 5′SOLiD, we compared the 5′SOLiD library to a 5′SAGE (19 base) library prepared from HT-29 cells treated with 5Aza. The 5′SAGE libraries comprised approximately 70,000 tags (Table S2). The sensitivity of the 5′SAGE method appeared to be equivalent to the original SAGE method. Figure 2 shows scatter plots comparing RNA levels determined using the 5′SOLiD and 5′SAGE systems. The X and Y coordinates indicate the number of 5′-end tag-associated genes identified within the control and 5Aza treated cells. A simple comparison of the scatter plots of the data generated from these two different methods, indicates that the dynamic range of the number of unique transcripts identified by 5′SOLiD per single run is 100–1,000 fold greater than that observed for 5′SAGE (Figure 2, Table 3). However, the number of 5′SOLiD tags identified per library sample is one hundred-fold greater than the number of 5′SAGE tags detected per library sample. Therefore to subtract out the differential sensitivity caused by the significantly greater number of SOLiD tag reads relative to the number of capillary tag reads, we selected a random one percent of the 5′SOLiD tag reads so that the selected 5′SOLiD tags were roughly equivalent in number to the 5′SAGE tags. Figure 2b displays the scatter plot of this selected subset of 5′SOLiD tags. The Pearson coefficients of the genes from the selected 5′SOLiD tags were greater than those observed for the 5′SAGE tags, indicating that the 5′SOLiD tags are more likely to fit to the diagonal. In addition, the MA plots generated from each scatter plot are shown in Figures 2d–f. In order to display the wide dynamic range of a method, an MA plot is preferred to a scatter plot because points are depicted along the horizontal line rather than the diagonal line. As indicated, the larger variance relative to 5′SAGE is more easily observed than when using scatter plot analysis for both the 5′SOLiD and 5′SAGE. It shows graphically how the dynamic range of the 5′SOLiD is approximately 100 fold greater than that of the 5′SAGE. More specifically, most of data plotted in Figures 2d (5′SOLiD) and 2f (5′SAGE) fall within the red regions, an index to compare the dynamic range. Observe that the width of this red region in the 5′SOLiD data plot is 2^(16−4)^ = 1024, while that of the 5′SAGE plot is only 2^(10−4)^ = 32. It is also remarkable that the distribution of data points within the red regions are less broad when using the 5′SOLiD compared to the distributions of the data points when using the 5′SAGE.

**Table 3:** Comparison of cell-cycle related gene profiling between 5′-end SAGE and 5′-end-SOLiD.

Description	5′-end SAGE (n)		RefSeq	5′endSOLiD (n)	
	Cont	5Aza		Cont	5Aza
eukaryotic translation elongation factor 2	33	7	NM_001961	5605	4110
cyclin-dependent kinase inhibitor 1A	0	1	NM_000389	34	55
cyclin-dependent kinase inhibitor 2A	0	2	NM_000077	172	124
cyclin-dependent kinase inhibitor 1B	0	0	NM_004064	61	59
cyclin-dependent kinase inhibitor 2B	0	0	NM_004936	27	70
cyclin-dependent kinase inhibitor 1C	0	0	NM_000076	6	6
cyclin-dependent kinase inhibitor 2C	0	0	NM_001262	124	109
cyclin-dependent kinase inhibitor 2D	0	1	NM_001800	319	248
cyclin-dependent kinase inhibitor 3	1	0	NM_005192	477	591
cyclin-dependent kinase 2	0	0	NM_001798	384	406
cyclin-dependent kinase 3	0	0	NM_001258	34	24
cyclin-dependent kinase 4	7	4	NM_000075	1077	1155
cyclin-dependent kinase 5	0	0	NM_004935	998	996
cyclin-dependent kinase 6	0	0	NM_001259	459	406
cyclin-dependent kinase 7	0	1	NM_001799	162	118
cyclin-dependent kinase 8	0	0	NM_001260	78	63
cyclin-dependent kinase 9	0	0	NM_001261	259	199
cyclin-dependent kinase 10	0	1	NM_052988	183	161
retinoblastoma 1	0	0	NM_000321	71	59
tumor protein p53	0	3	NM_000546	1068	1260
myc proto-oncogene protein	0	0	NM_002467	337	340
MAX protein isoform f	0	0	NM_197957	73	76
adenomatosis polyposis coli	0	0	NM_000038	156	138
phosphatase and tensin homolog	0	0	NM_000314	245	223
matrix metalloproteinase 7	7	6	NM_002423	4458	3800
erbB-2	0	1	NM_001005862	412	349
erbB-3	0	1	NM_001982	1046	851
v-erb-a erythroblastic leukemia viral oncogene	0	0	NM_001042599	171	84
wee1 tyrosine kinase	0	0	NM_003390	277	204
F-box only protein 5	0	0	NM_012177	48	41
cyclin A	0	1	NM_001237	657	617
cyclin B1	0	0	NM_031966	1144	1587
cyclin B2	0	0	NM_004701	975	686
cyclin D1	1	3	NM_053056	311	449
cyclin D2	0	0	NM_001759	3	3
cyclin D3	0	0	NM_001760	225	220
cyclin E1	0	0	NM_001238	83	66
cyclin E2	0	0	NM_057749	17	13
cyclin J	0	0	NM_019084	43	44
cyclin J-like	0	0	NM_024565	133	141
cyclin M2	0	0	NM_017649	53	58
cyclin M4	0	0	NM_020184	48	56
cyclin N-terminal domain containing 2	0	0	NM_024877	6	11
cyclin T1	0	0	NM_001240	15	14
cyclin Y-like 1	0	0	NM_152523	32	31
cell division cycle 25A	0	0	NM_001789	306	348
cell division cycle 25B	0	0	NM_004358	907	867
cell division cycle 25C	0	0	NM_001790	177	120
jun oncogene	0	0	NM_002228	670	626
polo-like kinase	0	1	NM_005030	414	612
secreted frizzled-related protein 1	0	0	NM_003012	6	2
secreted frizzled-related protein 5	1	0	NM_003015	1	1
secreted frizzled-related protein 2 precursor	0	0	NM_003013	1	0
myeloid cell leukemia sequence 1	0	0	NM_021960	1106	951
cell division cycle associated 3	0	0	NM_031299	44	43
catenin (cadherin-associated protein), beta 1,	0	0	NM_001098209	424	499
tumor protein p73	0	0	NM_005427	25	14
cadherin 1, type 1 preproprotein	1	0	NM_004360	1515	1428
lymphoid enhancer-binding factor 1	0	0	NM_016269	21	10
glycogen synthase kinase 3 beta	1	0	NM_002093	436	489

In this table, each number of tags from 5′SAGE, 5′SOLiD was normarized to 40,000 and 6,000,000.

We determined that the expression of 277 genes differed significantly between the control and 5Aza-treated cells, using the criteria of a fold change ≥2 and a Bonferroni adjusted *p-value* <0.001. The number of these differentially expressed genes corresponds to 1.8% (in 5Aza) of all expressed genes in a single cell type. In contrast, 5′SAGE identified only a few genes exhibiting significant changes in expression levels. Only 41 genes (Bonferroni adjusted *p-value* <0.001 and greater than 2-fold difference) exhibited a significant difference in expression between control and 5Aza-treated cells.

Table 3 compares the expression of cell cycle-related genes by gene profiling using the 5′SAGE and 5′SOLiD methods. As predicted by the scatter plots of the data generated by the two methods, most cell growth-related genes were present below the limits of detection of 5′SAGE. In contrast, most of these genes were detected with a sensitivity of between 100 to 1,000 tags on the 5′SOLiD platform. For example, whereas the myc proto-oncogene was not detected by 5′SAGE analysis (tag count: cont, 0; 5Aza, 0), it was easily detected using the 5′SOLiD method (cont, 337; 5Aza, 340) (Table 3).

### The differentially expressed genes in epigenetic drug-treated cells

Genes whose expression was altered by greater than 20 fold in response to exposure of cells to 5Aza relative to the control cells are listed in Table S3. A greater number of these genes exhibited an increase rather than a decrease in expression in response to 5Aza.

We next used the Gene Ontology Consortium database to categorize the function of those highly expressed genes whose expression was altered by greater than a 10 fold change in response to 5Aza treatment (Table S4). We observed that genes involved in the immune response, defense response and chemotaxis were differentially expressed in 5Aza-treated cells.

### Validation of the data using real-time PCR with TaqMan probes

To confirm the results of the 5′SOLiD analysis, we performed quantitative real-time PCR (qPCR) on forty transcripts identified in the previous section. We observed a good correlation between the changes in gene expression in response to 5Aza treatment detected b**y** 5′SOLiD data and qPCR. T and regression analyses between the two methods revealed a high level of consistency between the two sets of data (Pearson's correlation coefficient *r* = 0.985) (Figure 1d). Figure S2 shows nine representative examples of the correlation between the 5′SOLiD tags and qPCR. It is clear from this analysis that the results of 5′SOLiD method correspond very well to those of qPCR, even for genes expressed at only low copy numbers.

### Distribution of 5′SOLiD tags within known genes

**Figure 3:**
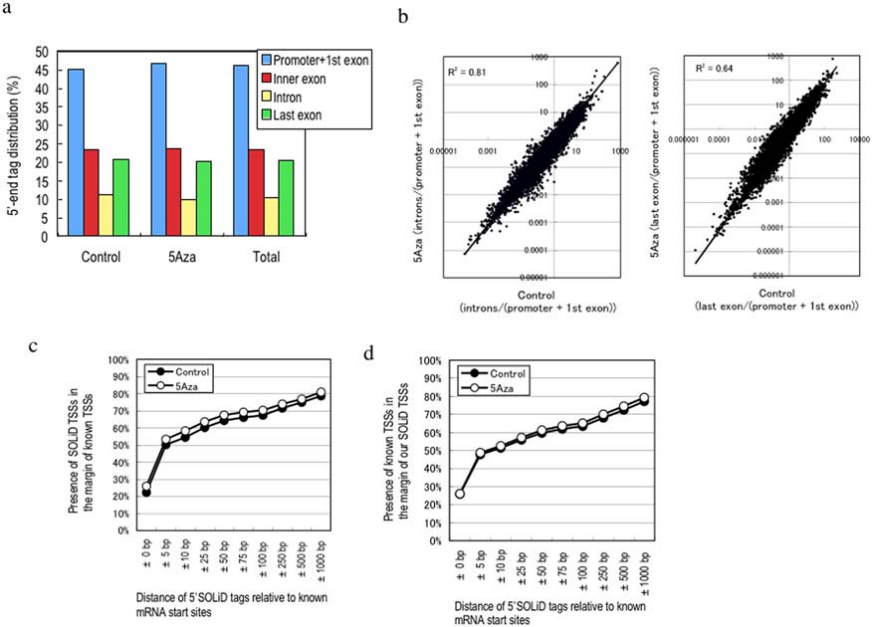
The distribution of 5′-end tags that correspond to annotated exons and introns of well-characterized genes(a). (b); Correlation of fold change of the number of tags within the promoter+1st exon relative to the number of tags in the inner exon/intron. The solid lines represent a linear regression fit. The slope is 0.79, 0.73 for the control versus 5Aza (intron/promoter+1st exon) or the control versus 5Aza (exon)/promoter+1st exon), respectively. (c) and (d). Analysis of TSS identified by SOLiD sequence tags. (c) Ratio of 5′SOLiD tags from each library to known TSSs and all libraries. (d) Ratio of SOLiD TSSs to known 5′end tags. Horizontal axes shows the distance of 5′SOLiD tags relative to the mRNA start sites in 1,562,911 known TSSs collected from a variety of human tissues in DBTSS database. Distances are shown as the number of upstream and downstream nucleotides. The coverage is given on the y-axis.

5′SOLiD tags identify the 5′-end of transcripts. Therefore, we analyzed the distribution of 5′-end tags annotated in exons/introns of known transcripts. Although the highest tag density occurs in the canonical position around the 5′-end of well-characterized genes or in the upstream regions of defined TSSs in the databases, some tags were localized to introns, inner exons or proximal to the most 3′ exon (Figure 3a).

To evaluate whether the 5′-end tags within a given gene region are expressed independently, we performed regression analysis of the fold differences between the numbers of tags localized to the promoter+1st exon relative to the number localized to inner exons/introns in the Control and 5Aza -treated libraries. Each linear regression analysis indicated a good fold-change correlation (Figure 3b). This result indicates that the expression of 5′-end tags in the same gene region correlates with those in the other exons and introns. These results suggests that the transcripts within a given gene region are coordinately perturbed by 5Aza.

### Coverage of known TSSs by 5′SOLiD tags

We next evaluated the number of 5′SOLiD tags that were represented among the known TSS in the DBTSS database, which contains the largest collection of human TSSs, and conversely we evaluated what fraction of the TSS contained with the DBTSS were represented within the 5′SOLiD TSSs data. Figure 3c shows that 80% of the 5′-end tags present within the 5′SOLiD dataset mapped to within 1,000 bp upstream or downstream of the 1,562,911 known TSSs within the DBTSS, an area which comprises only 4.3% of the genome. The rest are potentially novel TSSs revealed by the enormous collection of 5′end tags. Figure 3d shows that only 80% of the known TSSs in the DBTSS are represented within the 5′SOLiD TSSs tags. The remaining 20% of known TSSs in the DBTSS may be derived from many different tissues and samples, which may explain why we did not detect them within the particular cell type used in this study. In addition, the diversity of the transcription start sites was not changed by treatment of cells with 5Aza.

## Discussion

Massively parallel, tag-based sequencing systems, such as the SOLiD system, hold the promise of revolutionizing the study of whole genome gene expression due to the number of data points that can be generated in a simple and cost-effective manner. We describe here the development of a 5′-end sequencing method, 5′ SOLiD, and demonstrate the advantages offered by this tag-based system with regards to sensitivity and dynamic range over traditional approaches to studying whole genome gene expression. Recently, various studies using next generation sequencing to evaluate transcriptional factor binding sites [19], histone modifications [6], DNase I hypersensitive sites [20], and nucleosome positions [7], [10] have discussed the relative distance between TSSs and these examined sites. However, the TSS information used in these analyses was downloaded from known database and was not based on the use of real TSSs identified within each examined library. In addition, while several technologies for RNA sequencing of whole transcripts have been reported, large-scale sequencing is still required. These problems are overcome by our method. The 5′SOLiD method enables cost-effective high-throughput genome-wide identification of TSSs and analysis of gene expression of low-copy mRNAs in a comprehensive fashion (Table S5).

## Materials and Methods

### 5Aza-treatment, and RNA preparation

HT-29 cells were cultured in RPMI1640 or McCoy's 5A medium supplemented with 10% fetal bovine serum. Cells were incubated with 5Aza (Sigma) (500 nM) for 72 h; the culture medium was replaced with fresh medium with 5Aza every 24 h. Total RNA was extracted using RNA-Bee prior to analysis by 5′SAGE and RT-PCR. The quality of total RNA was analyzed using an Agilent 2100 Bioanalyzer. Prior to 5′SAGE analysis and 5′SOLiD, we examined the influence of 5Aza on HT-29 cells using bisulfite-PCR to determine the methylation status of CpG islands in the MAGE-1 promoter. We found decreased methylation in the MAGE1 promoter after 5Aza-treatment in HT-29 cells. This preliminary experiment confirmed that epigenetic changes were induced by 5Aza (data not shown).

### Generation of 5′SAGE library

5′SAGE libraries were created as previously described [4]. Five to ten micrograms of poly(A)+RNA was treated with bacterial alkaline phosphatase (BAP; TaKaRa). The poly(A)+RNA was extracted twice with phenol∶chloroform (1∶1), ethanol precipitated, and then treated with tobacco acid pyrophosphatase (TAP) . Two to four micrograms of the BAP- TAP treated poly(A)+RNA were divided into two aliquots and an RNA linker containing recognition sites for EcoRI/MmeI was ligated using RNA ligase (TaKaRa): one aliquot was ligated to a 5′-oligo 1 (5′-GGA UUU GCU GGU GCA GUA CAA CGA AUU CCG AC -3′) linker, and the other aliquot was ligated to a 5′-oligo 2 (5′-CUG CUC GAA UGC AAG CUU CUG AAU UCC GAC -3′) linker. After removing unligated 5′-oligo, cDNA was synthesized using RNaseH free reverse-transcriptase (Superscript II, Invitrogen) at 12°C for 1 h and 42°C for the next hour, using 10 pmol of dT adapter-primer (5′-GCG GCT GAA GAC GGC CTA TGT GGC CTT TTT TTT TTT TTT TTT-3′).

After first-strand synthesis, RNA was degraded in 15 mM NaOH at 65°C for 1 h. cDNA was amplified in a volume of 100 ul by PCR with 16 pmol of 5′ (5′ [biotin]- GGA TTT GCT GGT GCA GTA CAA –3′) or (5′ [biotin]- CTG CTC GAA TGC AAG CTT CTG-3′ ) and 3′ (5′-GCG GCT GAA GAC GGC CTA TGT-3′) PCR primers. The cDNA was amplified using 10 cycles at 94°C for 1 min, 58°C for 1 min, and 72°C for 2 min. PCR products were digested with the MmeI type IIS restriction endonuclease (University of Gdansk Center for Technology Transfer). The digested 5′-terminal cDNA fragments were bound to streptavidin-coated magnetic beads (Dynal, Oslo, Norway). cDNA fragments that bound to the beads were directly ligated together in a reaction mixture containing T4 DNA ligase in a supplied buffer for 2.5 h at 16°C. The ditags were amplified by PCR using the following primers: 5′ GGA TTT GCT GGT GCA GTA CA 3′ and 5′ CTG CTC GAA TGC AAG CTT CT 3′. The PCR products were analyzed by polyacrylamide gel electrophoresis (PAGE) and digested with EcoRI. The region of the gel containing the ditags was excised and the fragments were self-ligated to produce long concatamers that were then cloned into the EcoRI site of pZero 1.0 (Invitrogen). Colonies were screened with PCR using the M13 forward and reverse primers. PCR products containing inserts of more than 600 bp were sequenced with the Big Dye terminator ver.3 and analyzed using a 3730 ABI automated DNA sequencer (Applied Biosystems, Foster City, CA). All electrophoretograms were reanalyzed by visual inspection to check for ambiguous bases and to correct misreads. In this study, we obtained 19–20 bp tag information.

### Generation of 5′SOLiD library

Five to ten micrograms of poly(A)+RNA was treated with bacterial alkaline phosphatase (BAP;TaKaRa). The poly(A)+RNA was extracted twice with phenol∶chloroform (1∶1), ethanol precipitated, and treated with tobacco acid pyrophosphatase (TAP). Then an RNA linker containing recognition sites for EcoP15I was ligated using RNA ligase (TaKaRa): a 5′-oligo 1 (5′- CUG CCC CGG GUU CCU CAU UCU CU CAG CAG -3′) linker. After removing unligated 5′-oligo, cDNA was synthesized using RNaseH free reverse-transcriptase (Superscript II, Invitrogen) at 12°C for 1 h and 42°C for the next hour. A 5′-end- cDNA library was produced using 10 pmol of dT EcoP adapter-primer (5′- GCG GCT GAA GAC GGC CTA TGT GCA GCA G(T)_17_ -3′).

After first-strand synthesis, RNA was degraded in 15 mM NaOH at 65°C for 1 h. cDNA was amplified in a volume of 100 ul by PCR using 16 pmol of 5′ (5′ [biotin]- CTG CCC CGG GTT CCT CAT TCT –3′) and 3′ (5′- GCG GCT GAA GAC GGC CTA TGT -3′) PCR primers. The cDNA was produced using 10 cycles at 94°C for 1 min, 58°C for 1 min, and 72°C for 10 min.

PCR products were digested with the EcoP15I type IIS restriction endonuclease (NEB). The digested 5′-terminal cDNA fragments were bound to streptavidin-coated magnetic beads (Dynal, Oslo, Norway). cDNA fragments that bound to the beads were blunted and then directly ligated to the SOLiD linker in a reaction mixture containing T4 DNA ligase in the supplied buffer for 2.5 h at 16°C. The samples were amplified by PCR using the primers: 5′-CCACTACGCCTCCGCTTTCCTCTCTATG-3′ and 5′-CTGCCCCGG GTTCCTCATTCT-3′. The PCR products were purified by polyacrylamide gel electrophoresis (PAGE). The purified libraries were sequenced with the SOLiD system according to the manufacturers' protocol (Applied Biosystems, Foster City, CA). We confirmed the integrity of the cDNA using an Agilent 2100 Bioanalyser prior to construction of the 5′SOLiD libraries.

### SOLiD tag mapping

In the SOLiD Sequencing System, after each cycle of ligation, the color that is detected represents 4 potential two base combinations and the conversion into nucleotide base space is usually done after the sequence is aligned to the reference genome translated into in the colorspace coding. This two base encoding strategy provides higher sequencing accuracy and inherent error checking capability. The details of principles of SOLiD sequencing and advantages of colorspace are summarized at the link below. http://marketing.appliedbiosystems.com/images/Product_Microsites/Solid_Knowledge_MS/pdf/SOLiD_Dibase_Sequencing_and_Color_Space_Analysis.pdf. The algorithm “SOLiD™ System Color Space Mapping Tool (mapreads)” uses spaced seeds for high speed and sensitive global alignment. It transforms the reference to colorspaces then maps the reads. The opposite transformation that changes colorspace to dnaspace (or basespace) should be avoided because a single mismatch in colorspace will affect all the following base calls. In our report, a mismatch means a colorspace differs between a read and the reference. The software can be downloaded from http://solidsoftwaretools.com/gf/project/mapreads/. SOLiD tags were mapped to the human genome (hg17) using the mapping tool developed at ABI. The first 25 color spaces of tags were used for alignment allowing up to two mismatches. “Unique tags”, whose best sequence matches were located on unique positions in the genome, were used in subsequent tag assignment analysis for high accuracy. Unique tags that were aligned with the same start position on the same strand of the genome were treated as identical even if they contained at most two mismatches relative to the genome sequence.

### Assignment of SOLiD Tags

We associated a unique tag with a known transcription start site (TSS) registered in the DBTSS (ver5.2, http://dbtss.hgc.jp/) if the start position of the tag was located within the *±n* bp of the TSS in the genome. Similarly, we assigned a unique tag to a RefSeq gene (UCSC, http / hgdownload.cse.ucsc.edu /goldenPath/hg17/database/) if the start position of the tag fell within a range from 500 bp upstream of the TSS to the end of the RefSeq gene. For further analysis, a unique tag was associated with a transcript annotation based upon being mapped within the promoter, an exon, or an intron of a RefSeq gene.

### Real-time PCR

cDNA was prepared from total RNA using the Applied Biosystems cDNA archive Kit and random primers. Multiple reactions containing 10 ug total RNA per 100 ul reaction volume were performed for each sample following the manufacturer's recommendations. TaqMan gene expression assays were performed using a FAM-labeled MGB probe. Each TaqMan assay was performed in triplicate for each RNA sample. 7.5 ng total cDNA(as total input RNA) in a 15 ml final volume was used for each assay. Assays were performed with 2xUniversal Master on Applied Biosystems 7500 (Foster City, CA) using universal cycling conditions (2 min at 50°C, 10 min at 95°C, followed by 40 rounds of 15 s at 95°C and 1 min at 60°C). G3PDH was chosen as the reference gene.

#### Statistical analysis

Comparison of each 5′SOLiD tag was calculated using Z-test statistics[21]. The difference of expression proportions *p*_1_ and *p*_2_ , resulting from samples with sizes *n*_1_ and *n*_2_ respectively, is calculated by:
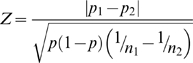
where *p* is calculated as 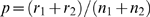, which is the estimate of the proportions *n*_1_ and *n*_2_ respectively if the null hypothesis were true, where *r*_1_ and *r*_2_ are expressed tags for a gene in the sample. Under the null hypothesis this *Z* statistics is normally distributed and can server as a statistical test for the difference between the proportions *p*_1_ and *p*_2_. For each comparison group (e.g. control-5Aza), we evaluated genes that were expressed in both samples. If given gene was not represented by a transcript (the number of transcripts from a certain gene was calculated from the number of tags observed in the respective sample), we did not include them in the gene set used to perform the Z-test. In this way, we observed 15,439 genes in the control-5Aza group. After the Bonferroni adjustment, we fixed p-value <0.001 for the aforementioned Z-test in our differential expression analysis.

### Accession number

5′SOLiD tags have been deposited at NCBI Short Read Archive under the project accessions SRA002659.

## Supporting Information

Figure S1 5′SOLiD is highly reproducible.
(0.05 MB PDF)

Figure S2 Validation of 5′SOLiD by quantitative real-time PCR using TaqMan probes.
(0.09 MB PDF)

Table S1 Transcript profile in HT29 treated with 5Aza.
(0.02 MB XLS)

Table S2 5′SAGE tag mapping summary
(0.02 MB XLS)

Table S3 Differentially expressed genes in cells treated with 5Aza
(0.02 MB XLS)

Table S4 Gene Ontology Comparison
(0.02 MB XLS)

Table S5 Cost- and time-benefit analysis of 5′SOLiD, 5′SAGE and SAGE
(0.03 MB XLS)
