# Supplementary material for: High-Resolution Analysis of the 5′-End Transcriptome Using a Next Generation DNA Sequencer
Source: PLoS One. 2009 Jan 1;4(1):e4108. doi: 10.1371/journal.pone.0004108 (PMC2606021; doi:10.1371/journal.pone.0004108)
Supplement: Figure S1 — 5′SOLiD is highly reproducible. (0.05 MB PDF) [file pone.0004108.s001.pdf]

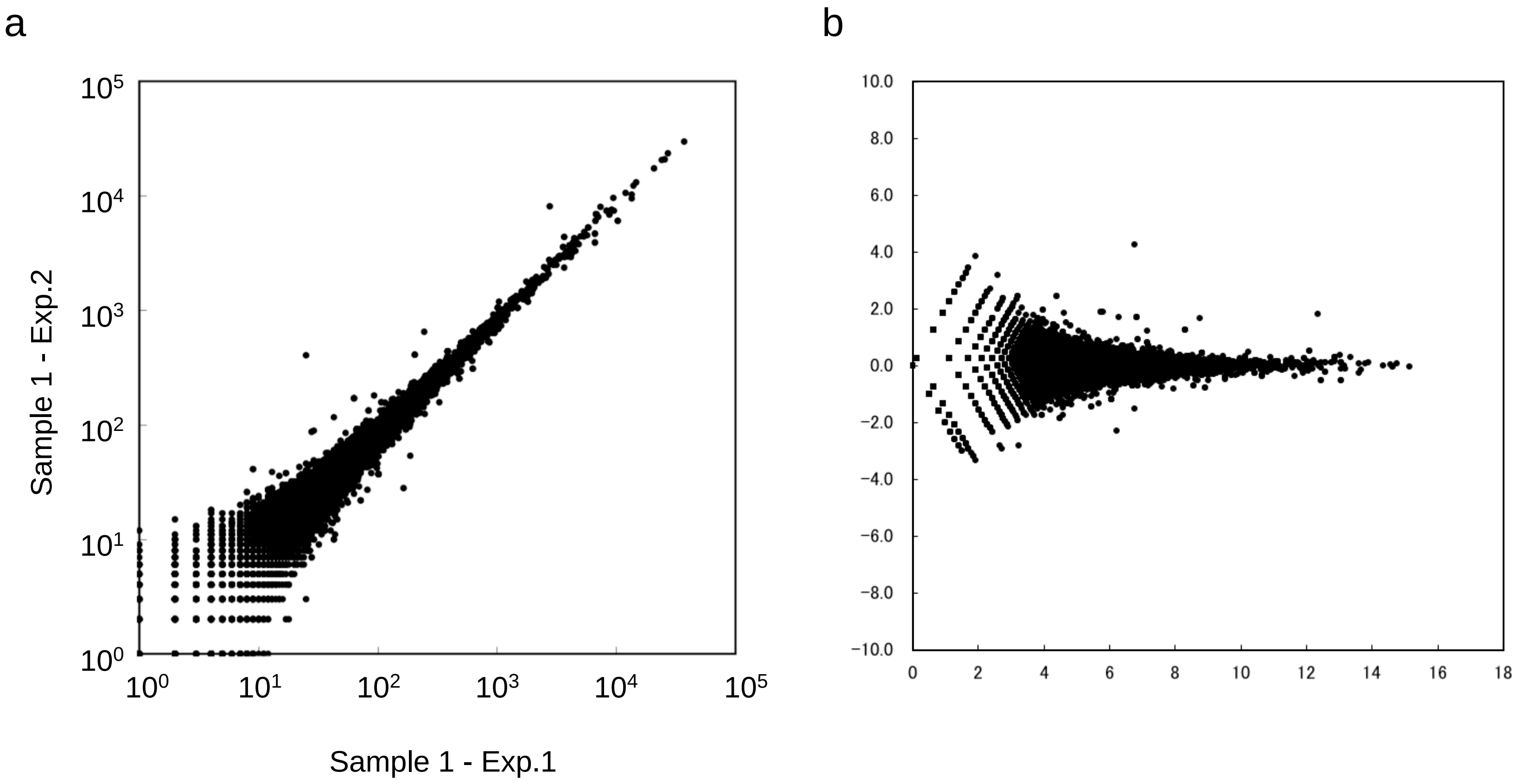

Figure S1. 5'SOLiD is highly reproducible. These data shows the reproducibility of unique transcripts identified from SOLiD sequencing of libraries. Individual 5'-end tag is associated with the human gene in which the tag originated. In the graph, each dot represents one gene, and its x and y coordinates indicate the numbers of 5'-end tags associated with the gene in each experiment (a). A Pearson coefficient (r) of >.99 was calculated, illustrating a strong correlation between the results of the two independent runs. Number of 5'-end tags were from sample 1- Exp.1, 2,388,068, Exp.2 1,992,160. (b) MA plots are calculated from the pair of the number of Exp.1 and Exp.2, that are associated with a gene. X and Y values are translated into A and M values according to the following formulas: $A = \frac{1}{2}(\log_2 X + \log_2 Y)$, $M = \log_2 X - \log_2 Y$.
