# Supplementary material for: High-Resolution Analysis of the 5′-End Transcriptome Using a Next Generation DNA Sequencer
Source: PLoS One. 2009 Jan 1;4(1):e4108. doi: 10.1371/journal.pone.0004108 (PMC2606021; doi:10.1371/journal.pone.0004108)
Supplement: Figure S2 — Validation of 5′SOLiD by quantitative real-time PCR using TaqMan probes. (0.09 MB PDF) [file pone.0004108.s002.pdf]

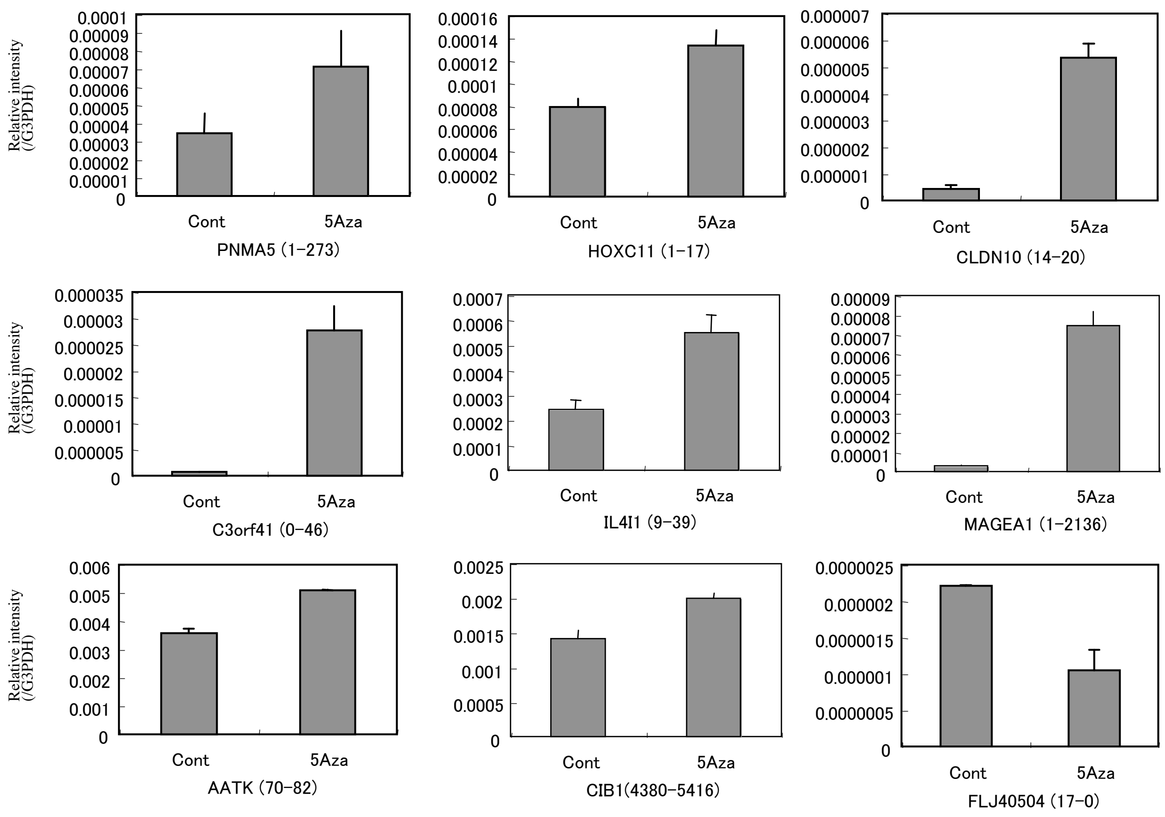

Figure S2. Validation of 5'SOLiD by quantitative real-time PCR using TaqMan probes. The increased and decreased genes in 5Aza treated cells were evaluated. Each number in a parenthesis represents the number of 5'SOLiD tags from each library. Each number of tags was normalized to 6,000,000.
